# CONFERENCE ON STANDARDS AND TRADE

**DOI:** 10.6028/jres.092.035

**Published:** 1987-10-01

**Authors:** Walter G. Leight

**Affiliations:** Office of Product Standards Policy, National Bureau of Standards, Gaithersburg, MD 20899

The International Trade Administration and the National Bureau of Standards, of the Department of Commerce, sponsored a one-day Conference on Standards and Trade on May 5, 1987. The purpose of the Conference was to identify and explore possible actions by the Government and the private sector to enhance U.S. trade through promoting U.S. standards, and the technology they embody, in foreign countries and international organizations.

The Conference was co-chaired by the Honorable Bruce Smart, Under Secretary for International Trade, and Dr. Ernest Ambler, Director of the National Bureau of Standards. The first of three segments was comprised of presentations by officials of the U.S. Government: the Honorable Clarence Brown, Deputy Secretary of Commerce, the Honorable Michael Smith, Deputy U.S. Trade Representative, and Ambassador Diana Lady Dougan, U.S. Coordinator, Bureau of International Communications and Information Policy, U.S. Department of State. They stressed the need to improve the national trade picture, described ongoing efforts by the government under the GATT Agreement on Technical Barriers to Trade (the “Standards Code”), bilateral negotiations on standards-related trade problems, participation in treaty organizations and other government-to-government international committees, and the status of the current Uruguay Round of Multinational Trade Negotiations (MTN) talks. They expressed receptiveness to private sector views and their willingness to apply available resources to strengthen our trade position, especially in the international standards arena.

The second group of morning speakers addressed standards-related concerns of the business community. Dr. Peter Bell, Vice President of Corporate Technology, Norton Company, focused on export controls and expressed the desire for a single federal regulatory focus. Dr. Peter Bridenbaugh, Vice President for Research and Development, Alcoa, emphasized the need for U.S. leadership in technology and the development of the best technical standards possible. Mr. C. Scott Kulicke, Chairman and Chief Executive Officer Kulicke and Soffa Industries, Inc., described the activities of the Semiconductor Equipment and Materials Institute (SEMI) and the necessity for rapid development of new standards in a changing technological environment, as well as participation by foreign nationals in standards-developing bodies, both in the United States and elsewhere.

Private sector participation in international standardization was the subject of the third part of the morning session. Dr. Robert Baboian, Chairman of the ASTM Board, echoed and underscored the importance of using the best technical standards available as *de facto* international standards, ASTM’s role in international standards committee work, and in cooperating with the standards bodies of other countries. Dr. George S. Wham, Chairman, American National Standards Institute, described the private sector standards system and how ANSI functions as the member body to ISO and IEC.

Three Working Groups met in the early afternoon and submitted reports which may be summarized as follows:

## Participation in International Standardization Activities, Chair

Catherine Kachurik, CBEMA staff and Director, X-3 Secretariat. The private sector should seek funding by collections under a budgeted, equitable system or by special assessment. Hosting meetings in the United States would reduce costs to U.S. participants and benefit foreign counterparts through favorable exchange rates. The Group proposed media and educational campaigns to convince corporate executives and managers of the long-term value of participating in international standardization activities and to attract replacements for retiring “elder statesmen.” The Government should seek industry opinion, be responsive and reactive to standards needs, and support positions in GATT talks and other negotiations.

## Test Data Acceptance, Chair

Gerald Ritterbusch, Caterpillar, Inc. Initiatives to promote acceptance of test data by other countries should be pursued at all levels, including the current Uruguay Round of Multinational Trade Negotiations, bilateral negotiations, and the GATT Standards Code Committee. Voluntary laboratory accreditation programs, self-certification, third-party testing, and witnessed tests in the U.S. could reduce testing costs, with treatments matched to products and markets. An industry program should publicize certification programs, their costs, and significance.

## Adoption of U.S. Standards, Chair

Barbara Boykin, Aerospace Industries Association. Industry must “think internationally” about standards and trade, abandoning nationalistic approaches and adapting to the needs of international markets; strengthen representation in ISO and IEC to encourage adoption of U.S. standards; pursue harmonization efforts with the European Community; promote awareness and availability of U.S. standards to potential users abroad; educate U.S. Government officials overseas about standards; preserve private sector leadership and enhance government-industry cooperation.

Texts of presentations, summaries of question-and-answer periods, and reports of the Working Groups will be published in Conference Proceedings, available from the NBS Office of Product Standards Policy in July.

